# Atezolizumab-Induced Type 1 Diabetic Ketoacidosis in a Patient With Small Cell Lung Cancer and Pre-existing Type 2 Diabetes Mellitus

**DOI:** 10.7759/cureus.57024

**Published:** 2024-03-27

**Authors:** Kohei Narumoto, Naohiro Oda, Reo Mitani, Ichiro Takata

**Affiliations:** 1 Internal Medicine, Fukuyama City Hospital, Fukuyama, JPN

**Keywords:** diabetic ketoacidosis (dka), type 1 diabetes mellitus (t1d), immune-related adverse event (irae), immune-checkpoint inhibitor (ici), lung cancer

## Abstract

In this report, we present a case of a 70-year-old male with small cell lung cancer (SCLC) and pre-existing type 2 diabetes mellitus (T2DM) who developed type 1 diabetic ketoacidosis (DKA) following treatment with atezolizumab plus chemotherapy. Despite well-controlled T2DM with oral hypoglycemic agents, the initiation of immune checkpoint inhibitors (ICIs) led to rapid deterioration into insulin-dependent status due to ICI-induced type 1 diabetes mellitus (T1DM). Vigilant monitoring for hyperglycemia and timely intervention is crucial during ICI therapy, considering the potentially life-threatening complications. Although the patient achieved extended progression-free survival (PFS) post-treatment, re-administration of atezolizumab resulted in a bullous pemphigoid-like rash, necessitating discontinuation of the drug and corticosteroid treatment. The impact of recurring immune-related adverse events (irAEs) on treatment efficacy warrants further investigation.

## Introduction

Type 1 diabetes mellitus (T1DM) is a rare but significant endocrine immune-related adverse event (irAE) associated with immune checkpoint inhibitors (ICIs). The incidence of T1DM as an irAE is 0.2-1.4% [1]. ICI-induced T1DM is particularly concerning due to its potential for diabetic ketoacidosis (DKA), a life-threatening condition that can develop rapidly and is irreversible if not promptly managed.

Although there is increasing literature on ICI-induced T1DM in patients with lung cancer, the majority of cases reported involve patients with non-small cell lung cancer, with very few instances occurring in patients with small cell lung cancer (SCLC) [2,3]. The clinical landscape of T1DM in SCLC remains relatively uncharted owing to the paucity of reported cases. Notably, recent clinical trials have shown the rarity of T1DM as an irAE among patients with SCLC. The IMpower133 trial [4], evaluating atezolizumab, an anti-programmed death-ligand 1 (PD-L1) antibody, plus chemotherapy, and the CASPIAN trial [5], exploring durvalumab (an anti-PD-L1 antibody) plus chemotherapy, both in patients with extensive-stage SCLC, have reported that T1DM occurred in one case (0.5%) in IMpower133 and four cases (2.0%) in CASPIAN.

In this report, we present the case of a patient with SCLC and pre-existing type 2 diabetes mellitus (T2DM) who developed type 1 DKA following atezolizumab plus chemotherapy. While this case underscores the rapid deterioration of glycemic control in patients with T2DM treated with ICIs, necessitating vigilant monitoring for potential life-threatening DKA, it also highlights the challenges posed by recurring irAEs such as the bullous pemphigoid-like rash observed upon re-administration of atezolizumab.

## Case presentation

**Figure 1:**
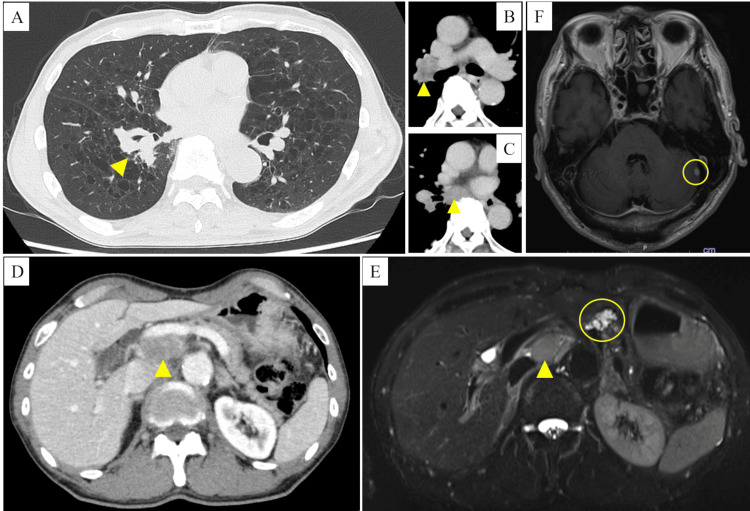
Contrast-enhanced computed tomography, magnetic resonance cholangiopancreatography, and gadolinium-enhanced head magnetic resonance imaging at diagnosis Contrast-enhanced computed tomography at diagnosis showing the tumor in the right lower lobe (yellow arrowhead) (A), right hilar and mediastinal lymphadenopathies (yellow arrowheads) (B, C), and intraabdominal lymphadenopathy (yellow arrowhead) (D). Magnetic resonance cholangiopancreatography showing intraabdominal lymphadenopathy (yellow arrowhead) and pancreatic cysts in pancreatic head (yellow circle) (E). Gadolinium-enhanced head magnetic resonance imaging showing the tumor in the left cerebellum (yellow circle) (F).

The patient was a 70-year-old male who visited our hospital for a close examination of an abnormal chest shadow. He was a former smoker (Brinkman index of 1,280), with T2DM, hypertension, and pancreatic cyst. His diabetes was well-controlled by treatment with an oral hypoglycemic agent (teneligliptin), with random plasma glucose of 174 mg/dL and HbA1c of 6.0%. Contrast-enhanced computed tomography showed the tumor in the right lower lobe (Figure 1A), right hilar and mediastinal lymphadenopathies (Figures 1B, 1C), and intraabdominal lymphadenopathy (Figure 1D). Magnetic resonance cholangiopancreatography showed no change in pancreatic cysts in the pancreatic head from previous imaging findings (Figure 1E). Gadolinium-enhanced head magnetic resonance imaging showed the tumor in the left cerebellum (Figure 1F).

**Figure 2:**
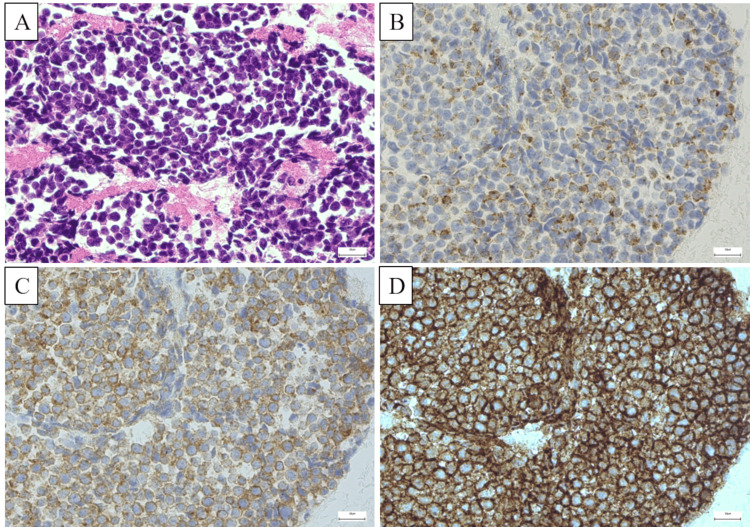
Histopathological findings Hematoxylin eosin staining shows small, round blue cells with scant cytoplasm and finely granular nuclear chromatin (A). Immunohistochemistry is positive for chromogranin A (B), synaptophysin (C), and CD56 (D).

We obtained a sample of tissues from the mediastinal and intraabdominal lymphadenopathies using endoscopic ultrasound with fine-needle aspiration. Histopathological examination results revealed small cell carcinoma (Figure 2).

**Figure 3:**
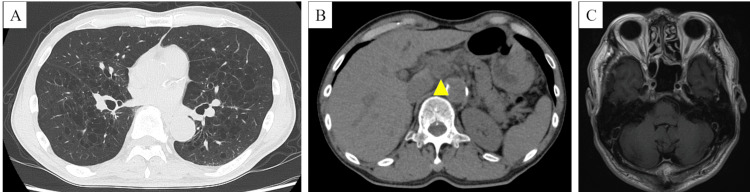
Computed tomography and gadolinium-enhanced head magnetic resonance imaging after the fourth course of carboplatin plus etoposide plus atezolizumab Computed tomography after the fourth course of carboplatin plus etoposide plus atezolizumab shows a decrease in the size of the tumor in the right lower lobe (A) and intraabdominal lymph node metastasis (yellow arrowhead) (B). Gadolinium-enhanced head magnetic resonance imaging shows the absence of left cerebellar metastasis (C).

He was diagnosed with a right lower lobe SCLC, cT1bN2M1c, stage IVB. He received four cycles of carboplatin plus etoposide plus atezolizumab and was partially responsive (Figure 3).

**Figure 4:**
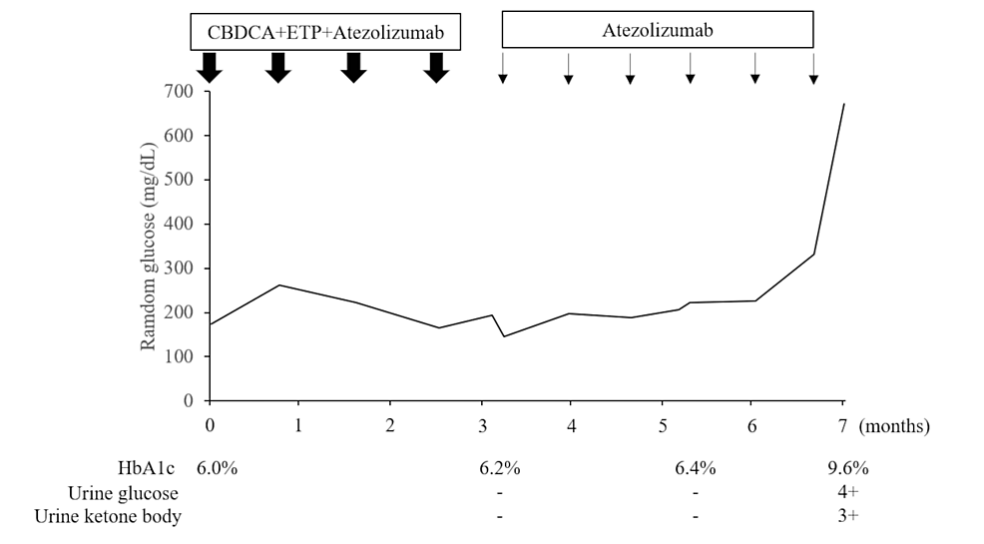
Clinical course CBDCA, carboplatin; ETP, etoposide

Maintenance therapy with atezolizumab continued thereafter and at the fourth cycle of atezolizumab, random plasma glucose was 222 mg/dL and HbA1c was 6.4% (Figure 4). After six cycles of atezolizumab, he had an urgent visit to our hospital because of thirst, anorexia, nausea, and vomiting continuously for three days.

Blood tests showed plasma random glucose of 672 mg/dL, HbA1c of 9.6% (Figure 3), total ketones of 13,498 µmol/L (acetoacetic acid 814 µmol/L and 3-hydroxyacetic acid 11,684 µmol/L), sodium of 132 mmol/L, potassium of 5.6 mmol/L, chloride of 92 mmol/L, blood urine nitrogen of 68 mg/dL, creatinine of 1.39 mg/dL, amylase of 282 U/L, and lipase of 90 U/L. Arterial blood gas analysis showed a pH of 7.029, pCO_2_ of 18.6 mmHg, and HCO_3_- of 7.2 mmol/L, indicating anion gap-opening metabolic acidosis. Urinalysis showed urinary glucose 4+ and ketone body 3+. Infusion of isotonic solution of 0.9% sodium chloride and continuous intravenous infusion of regular insulin was started as DKA in the high-care unit. On the second day of hospitalization, his blood glucose levels stabilized at 200 mg/dL. The fasting C-peptide levels were 0.13 mg/dL, and negative results were obtained for anti-glutamic acid decarboxylase (GAD) and anti-islet antigen-2 (IA-2) antibodies, after which subcutaneous insulin therapy was introduced. He was diagnosed with T1DM, which is an irAE of atezolizumab based on acute onset hyperglycemic symptoms, ketoacidosis, and low fasting C-peptide levels. After his glycemic control was stabilized by subcutaneous insulin therapy, atezolizumab maintenance therapy was resumed 33 days after the onset of DKA. After resuming seven cycles of atezolizumab maintenance therapy, he developed a bullous pemphigoid-like rash as an irAE, which improved after the discontinuation of atezolizumab and treatment with corticosteroids. Subsequently, 15 months after the initiation of carboplatin plus etoposide plus atezolizumab, the disease progressed with a single brain metastasis. He received stereotactic radiotherapy for brain metastasis followed by four cycles of carboplatin plus etoposide as a sensitive relapse.

## Discussion

This case shows a transition from well-controlled T2DM managed with oral hypoglycemic agents to insulin-dependent status due to the onset of T1DM induced by ICIs. Patients with T2DM experience deteriorating glycemic regulation when subjected to ICIs. It has been reported that the initiation of ICIs can cause a substantial decline in glycemic control among patients with pre-existing T2DM, with a notable percentage of 7.5-10.7% encountering an increase in glycemic control post-ICI commencement [6]. In cases where hyperglycemia arises during ICI administration in patients with pre-existing T2DM, it is imperative not to attribute it to incidental hyperglycemia or the customary deterioration of glycemic control in T2DM. Instead, a constant awareness of the potential onset of ICI-induced T1DM must be ensured. This is because ICI-induced T1DM, as exemplified in this case, may initiate rapid DKA. A systematic review of 172 cases revealed that 67.4% of patients developed fulminant DKA upon a diagnosis of ICI-induced T1DM [7]. According to the National Comprehensive Cancer Network (NCCN) guidelines for the management of immunotherapy-related toxicities of version 1.2024 [8], if fasting, random glucose, or both, exceed 250 mg/dL during ICI administration to a patient with a history of T2DM, a new onset of ICI-induced T1DM should be suspected and re-measuring serum glucose and measuring C-peptide should be considered, assessing DKA and evaluating autoantibodies. A diagnosis of ICI-induced T1DM can be established if C-peptide levels are low. However, pancreatic islet autoantibodies only turn positive in approximately half of cases with predominating anti-GAD antibodies [7]. Consequently, vigilant monitoring for alterations in glycemic control is imperative for patients with pre-existing T2DM undergoing ICI treatment, with appropriate interventions, including insulin therapy, tailored based on the severity of hyperglycemia and the presence of DKA [9].

The patient achieved partial response and progression-free survival (PFS) of 15 months after treatment with carboplatin plus etoposide plus atezolizumab, surpassing the median PFS of 5.2 months observed in the IMpower133 trial [4]. Existing literature suggests a correlation between the occurrence of irAEs and the efficacy of ICIs in non-small cell lung cancer [10]. In recent years, similar trends have been reported in the context of SCLC [11]. However, these reports did not include ICI-induced T1DM. The occurrence of ICI-induced T1DM itself is rare; therefore, its relevance to the anti-tumor effect is unclear, but appropriate management of ICI-induced T1DM could improve the prognosis of lung cancer.

The patient resumed atezolizumab after DKA had been mitigated and the blood glucose level had stabilized. However, he experienced a bullous pemphigoid-like rash that required the discontinuation of atezolizumab and corticosteroid treatment. Management of endocrine irAEs typically involves the resumption of normal hormonal function through appropriate hormone replacement therapy [12]. In an observational, cross-sectional, pharmacovigilance study of safety reports in the World Health Organization database VigiBase, 24,079 irAEs were evaluated, and when rechallenged with the same ICI, the recurrence rate of the initial irAEs was 28.8% and the occurrence of different irAEs was 4.4% [13]. The bullous pemphigoid-like rash required a relatively long-term corticosteroid treatment that impaired glycemic control. If an objective response to initial ICI therapy was achieved, the risk of toxicity on resumption may outweigh the benefit.

## Conclusions

ICI-induced T1DM can rapidly worsen glycemic control in patients with T2DM, necessitating close monitoring of blood glucose during ICI therapy. The potential for life-threatening DKA underscores the urgency of vigilance. Although the patient achieved extended PFS post-treatment, re-administration of atezolizumab resulted in a bullous pemphigoid-like rash, necessitating discontinuation of the drug and the initiation of corticosteroid treatment. The impact of recurring irAEs on treatment efficacy warrants further investigation.
